# Specificity of Hemodynamic Brain Responses to Painful Stimuli: A functional near-infrared spectroscopy study

**DOI:** 10.1038/srep09469

**Published:** 2015-03-30

**Authors:** Meryem A. Yücel, Christopher M. Aasted, Mihayl P. Petkov, David Borsook, David A. Boas, Lino Becerra

**Affiliations:** 1 MGH/HST Athinoula A. Martinos Center for Biomedical Imaging, Department of Radiology, Massachusetts General Hospital, Harvard Medical School, Charlestown, MA, USA; 2 Center for Pain and the Brain, Departments of Anaesthesia and Radiology, Boston Children's Hospital, Boston, MA; 3 Departments of Psychiatry and Radiology, MGH

## Abstract

Assessing pain in individuals not able to communicate (e.g. infants, under surgery, or following stroke) is difficult due to the lack of non-verbal objective measures of pain. Near-infrared spectroscopy (NIRS) being a portable, non-invasive and inexpensive method of monitoring cerebral hemodynamic activity has the potential to provide such a measure. Here we used functional NIRS to evaluate brain activation to an innocuous and a noxious electrical stimulus on healthy human subjects (n = 11). For both innocuous and noxious stimuli, we observed a signal change in the primary somatosensory cortex contralateral to the stimulus. The painful and non-painful stimuli can be differentiated based on their signal size and profile. We also observed that repetitive noxious stimuli resulted in adaptation of the signal. Furthermore, the signal was distinguishable from a skin sympathetic response to pain that tended to mask it. Our results support the notion that functional NIRS has a potential utility as an objective measure of pain.

Portable, robust, and reproducible methods of imaging pain could provide a basis for objective evaluation of pain. Prior studies of functional measures of brain activity have utilized fMRI, EEG, MEG and PET. While EEG and MEG measures the neuronal activity directly, fMRI and PET measures neuronal activity related localized hemodynamic and oxidative changes in brain. Near-infrared spectroscopy (NIRS) is a portable, non-invasive, inexpensive method of monitoring cerebral hemodynamic activity at moderate depths (surface cortices), which makes it suitable for studying pain^1^,^2^,^3^. NIRS is able to characterize the changes in concentrations of both oxygenated (HbO) and deoxygenated hemoglobin (HbR), which combined indicate change in total hemoglobin concentration (HbT). This is accomplished through the use of two wavelengths of near-infrared light, i.e. 690 nm and 830 nm lasers, and light sensitive photodiodes. Based on the change in intensity of both wavelengths, the change in each chromophore concentration can be calculated using the modified Beer-Lambert law^4^,^5^,^6^. By positioning NIRS optodes across relevant cortical volumes, it is possible to monitor the hemodynamic fluctuations caused by neuronal activity^7^. These hemodynamic fluctuations are the result of vascular dilation increasing cerebral blood flow to active areas of the brain. Therefore changes in HbO and HbT positively correlate with neuronal activity.

The brain response to evoked pain has been extensively studied over the last decades in health and disease (see reviews by Peyron et al., 2000^8^; Apkarian et al., 2005^9^). Several approaches have been used to induce noxious stimuli including thermal, mechanical, electrical, and chemical as well as modulation of the pain response by analgesics^8^,^9^. Here we utilized NIRS to evaluate brain activation to innocuous and noxious electrical stimuli. Electrical stimuli were used because they are less likely to produce skin sensitization^10^, they can be changed to provide activation in predominantly nociceptive and non-nociceptive fibers^11^, and may be more easily applied to patients in the operating room. We were interested in defining: (1) the specificity of the signal in brain regions responding to nociceptive electrical stimuli, specifically the primary somatosensory cortex; (2) whether the responses were physiologically resembling to reports using similar repetitive stimuli; and (3) whether NIRS could differentiate responses to innocuous and noxious stimuli. We hypothesized that following nociceptive system's stimulation, a NIRS signal will be observed in the primary somatosensory cortex (S1) contralateral to the stimulus^9^,^12^,^13^; that repetitive stimuli will show adaptation of the signal consistent with previous findings for noxious thermal stimulation^14^; that the signal will be distinguishable from a skin sympathetic response to pain^15^; and that the painful and non-painful stimuli can be segregated with this approach. Taken together, the specificity and sensitivity of the approach will provide a basis for evaluating responses to pain in drug trials, in the operating theater, and possibly in individuals who are otherwise unable to communicate.

## Results

### Specificity of the Nociceptive Responses

*Our data show three aspects of sensitivity and specificity to the noxious response*.

**Figure 1:**
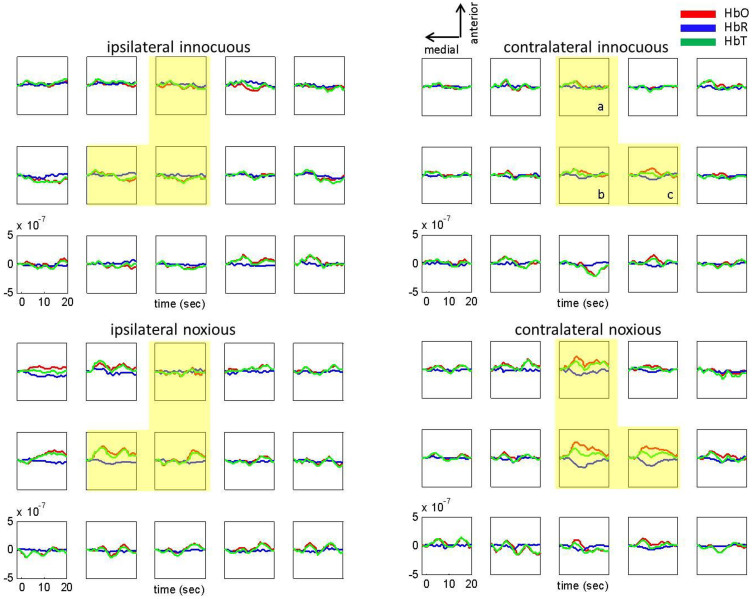
Localized hemodynamic response to left thumb innocuous and noxious electrical stimuli. Ipsilateral (left hemisphere) and contralateral (right hemisphere) hemodynamic response to left thumb innocuous (top panels) and noxious electrical stimuli (bottom panels). Group average results (n = 11) for the changes in HbO (red), HbR (blue), HbT (green). The letters a, b and c on the top right panel correspond to the same letters on Figure 7, panel B and Table 1.

**Figure 2:**
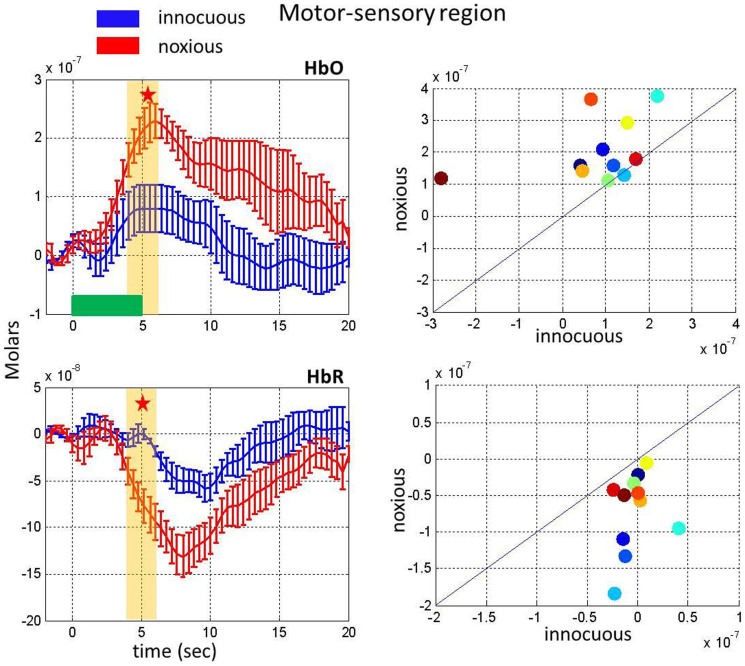
Comparison of the hemodynamic response to innocuous and noxious stimuli in the first three minutes on the motor-sensory region. Changes in HbO (top, left) and HbR (bottom, left) as a response to innocuous stimuli (blue) and noxious stimuli (red). Yellow bars show the interval chosen to obtain the mean responses depicted in the scatter plots and stars indicate a statistically significant difference. The right panels show a scatter plot comparing the hemodynamic response for each subject averaged over the yellow bar during the first three minutes for HbO (top) and HbR (bottom). The horizontal green bar shows the stimulus duration.

(1) Localized hemodynamic response to noxious electrical stimuli: Figure 1 shows the group averaged (n = 11) changes in HbO, HbR and HbT concentrations in response to innocuous and noxious electrical stimuli. Both stimuli resulted in a localized hemodynamic response in the right hemisphere (Figure 1, right panels), while noxious stimuli also produced a localized response in the left hemisphere. The average of the channels that showed the largest response to noxious stimuli (yellow area in Figure 1, right-bottom panel) was used in the rest of the analysis (please refer to Methods Section for probe placement). Table 1 provides the MNI coordinates of these channels, where the MNI coordinates were estimated using the procedure described by Tsuzuki et al., 2007^16^.The activation spans mainly the right post-central gyrus (two of the three channels of interest) where somatosensory cortex is located (Table 1).

(2) Noxious stimuli result in a greater response as compared to innocuous stimuli: The hemodynamic response to innocuous and noxious stimuli in the first three minutes was compared in Figure 2. The HbO response to noxious stimuli was significantly higher than the response to innocuous stimuli in the [4–6] sec time range (paired t-test, p = 0.008). We have chosen this time range since our stimulus lasts 5 seconds and we expect to see the peak response around 5 seconds. The magnitude of the HbR response to noxious stimuli was also significantly higher than the response to the innocuous stimuli (paired t-test, p = 0.002). The scatter plots show the robustness of this result for individual subjects (Figure 2, right panels).

**Figure 3:**
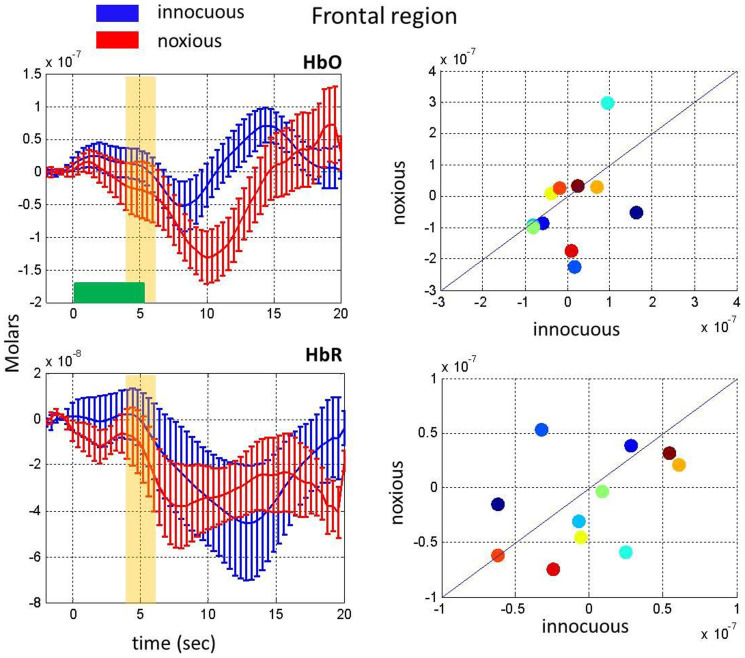
Comparison of the hemodynamic response to innocuous and noxious stimuli in the first three minutes on the frontal region. Changes in HbO (top, left) and HbR (bottom, left) as a response to innocuous stimuli (blue) and noxious stimuli (red). Yellow bars show the interval chosen to obtain the mean responses depicted in the scatter plots. The right panels show a scatter plot comparing the hemodynamic response for each subject averaged over the yellow bar during the first three minutes for HbO (top) and HbR (bottom). The horizontal green bar shows the stimulus duration.

The channels used for frontal region analysis cover the superior frontal gyrus and middle frontal gyrus (please refer to Methods Section for probe placement and channels of interest). Both innocuous and noxious stimuli resulted in a deactivation in the frontal channels as opposed to the motor-sensory region (Figure 3). The decrease in the signal in the [4–6] sec time range was not significantly different than baseline for both innocuous (paired t-test, p = 0.73 for HbO and p = 0.90 for HbR) and noxious (paired t-test, p = 0.51 for HbO and p = 0.36 for HbR). However, the signal change becomes significantly different than baseline in a later time range ([10–14] sec) for noxious (paired t-test, p = 0.04 for HbO and p = 0.03 for HbR). A trend toward significance was observed only for HbR during innocuous (paired t-test, p = 0.33 for HbO and p = 0.10 for HbR). Similarly neither HbO nor HbR response showed a statistically significant difference between an innocuous stimulus and a noxious stimulus in the [4–6] sec time range in the frontal region (paired t-test, p = 0.38 for HbO and p = 0.44 for HbR), however the difference becomes significant in the [10–14] sec time range for HbO (paired t-test, p = 0.05 for HbO).

**Figure 4:**
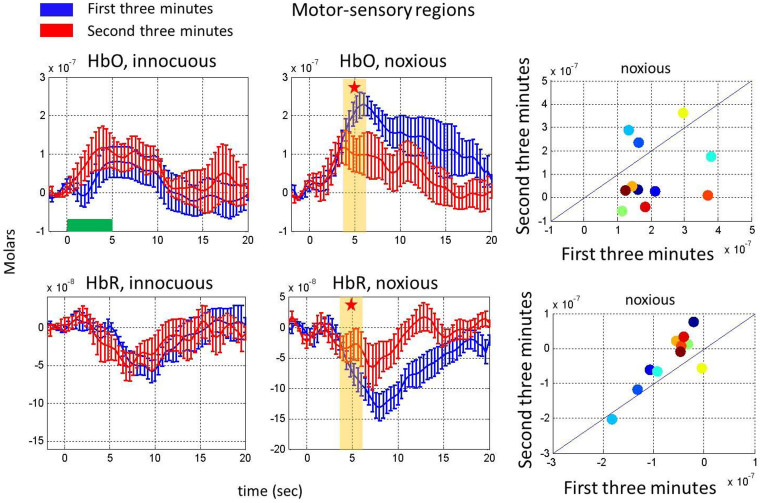
Habituation in the hemodynamic response to noxious stimuli on the motor-sensory region. Comparison of HbO (top, left panels) and HbR changes (bottom, left panels) in response to innocuous and noxious electrical stimuli in the first three minutes (blue) with the HbO and HbR changes in the second three minutes (red). Error bars represent the standard error across subjects (n = 11). Yellow bars show the interval chosen to obtain the mean response depicted for each subject in the scatter plots and stars indicate a statistically significant difference. The right panels show scatter plots comparing the hemodynamic response averaged over the yellow bar during the first and second three minutes for HbO (top) and HbR (bottom). The horizontal green bar shows the stimulus duration.

(3) Habituation in hemodynamic response to noxious stimuli: The group averaged changes in HbO and HbR concentrations in response to innocuous and noxious stimuli during the first three minutes and the second three minutes are compared in Figure 4 for the motor-sensory region. Note that in order to determine the time scale of the habituation effect, the data were divided into 6-minute, 3-minute and 2-minute epochs. From this analysis, we determined that the habituation effect was observable using 3 min epochs but not 2 min epochs with our stimulus presentation.

**Figure 5:**
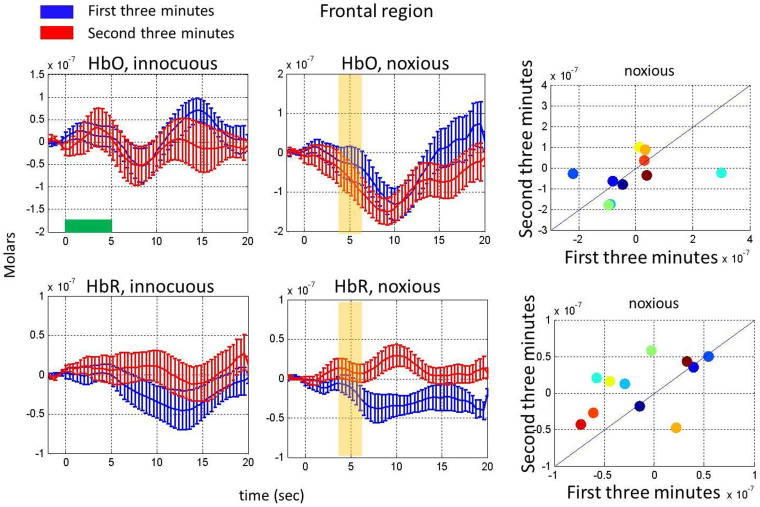
Habituation in the hemodynamic response to noxious stimuli on the frontal region. Comparison of HbO (top, left panels) and HbR changes (bottom, left panels) in response to innocuous and noxious electrical stimuli in the first three minutes (blue) with the HbO and HbR changes in the second three minutes (red). Error bars represent the standard error across subjects (n = 11). Yellow bars show the interval chosen to obtain the mean response depicted for each subject in the scatter plots and stars indicate a statistically significant difference. The right panels show scatter plots comparing the hemodynamic response averaged over the yellow bar during the first and second three minutes for HbO (top) and HbR (bottom). The horizontal green bar shows the stimulus duration.

The changes in HbO and HbR in response to noxious stimuli significantly decreased in the second three minutes as compared to the first three minutes of the experiment (paired t-test, p = 0.05 for HbO and p = 0.01 for HbR). There was no significant difference in the hemodynamic response to innocuous stimuli from the first to the second three-minute period (paired t-test, p = 0.66 for HbO and p = 0.22 for HbR). We also checked the robustness of our results for each subject. The scatter plots depict the response from each subject. Almost all the subjects had this habituation behavior (see scatter plots in Figure 4). The changes in HbO and HbR in response to noxious stimuli showed a similar habituation pattern in the frontal region, however the results were not statistically significant in the [4–6] sec time range (Figure 5) (paired t-test, p = 0.40 for HbO and p = 0.11 for HbR for the noxious stimulus and p = 0.99 for HbO and p = 0.76 for HbR for the innocuous stimulus).

We have performed a similar analysis to compare the 1^st^ three minute epoch to the 3^rd^ and 4^th^ three minute epochs. Our statistical analyses show a significant difference between the 1^st^ and 3^rd^ three minute epochs (paired t-test, p = 0.01), as well as between the 1^st^ and 4^th^ three minute epochs (paired t-test, p = 0.006). While we have observed a decrease in the response from 2^nd^ to 3^rd^ as well as from 3^rd^ to 4^th^ epochs, the difference was not found to be significant in the [4–6] sec time range.

(4) Bilaterality of the brain response to noxious stimuli: Figure 1 depicts the group averaged (n = 11) changes in HbO, HbR and HbT concentrations on both the ipsilateral and contralateral hemisphere in response to innocuous and noxious electrical stimuli. While no significant change in hemodynamic response was observed for the innocuous stimuli on the ipsilateral side (Figure 1, top, left panel), noxious stimuli triggered a brain activity on both sides (Figure 1, bottom panels). The average HbO and HbR responses (of the channels in yellow area in Figure 1) were significantly higher than baseline for the noxious stimuli on both hemispheres (paired t-test, contralateral hemisphere: p < 10^−4^ for HbO and p = 0.002 for HbR; ipsilateral hemisphere: p = 0.08 for HbO and p = 0.03 for HbR). Moreover, the strength of the response on the contralateral side is significantly higher than the response on the ipsilateral side on the highlighted channels (paired t-test, p = 0.01). A trend toward significance was observed only for HbO on the contralateral side for the innocuous stimuli (paired t-test, contralateral hemisphere: p = 0.08 for HbO and p = 0.46 for HbR; ipsilateral hemisphere: p = 0.69 for HbO and p = 0.16 for HbR).

### Sympathetic Skin Response vs. Brain Response

**Figure 6:**
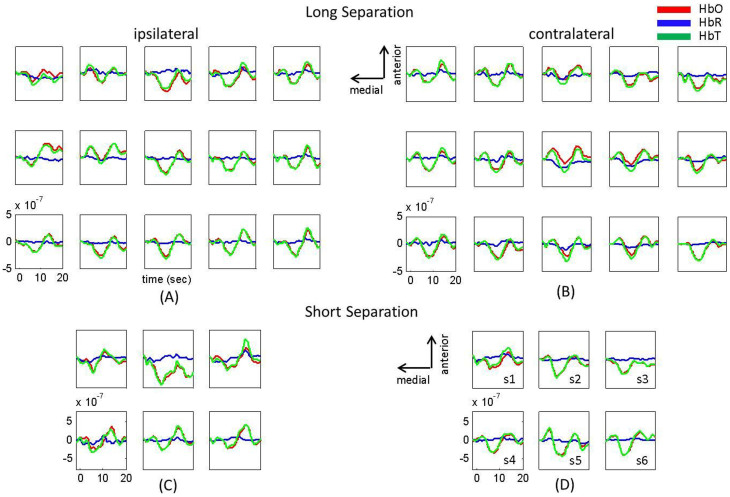
Hemodynamic response to left thumb noxious electrical stimuli at long separation channels (panels A and B) and short separation channels (panels C and D) without short separation regression. Group average results (n = 11) for the changes in HbO (red), HbR (blue), HbT (green). The letter-number combinations on panel D (from s1 to s6) correspond to the same letter-number combinations on Figure 7, panel B.

Figure 6 depicts the group averaged (n = 11) hemodynamic response to left thumb noxious electrical stimuli at short separation channels and at long separation channels using our general linear model analysis without the short separation regression. The long separation channel responses are different than the ones obtained using short separation regression (See Figure 1). This result shows that the pain stimuli trigger both a sympathetic skin response, as revealed by the short-separation responses seen in Figure 6, as well as a brain response (Figure 1). Using measurements that include short separation channels is important for regressing the superficial signal to permit more accurate estimate of the brain response to the noxious stimulus.

## Discussion

### Overview of Results

The results from this study indicate that electrical stimuli produce a well-localized brain response in primary somatosensory cortex as observed with other noxious stimuli^14^. However, since pain produces an autonomic response^10^, could the observed changes be a result of alterations in sympathetic control of blood flow? Noxious electrical stimulation results in both a sympathetic skin response and a brain response. The signal decrease observed at short separation channels is in agreement with literature that shows a significant decrease in skin perfusion after pain stimulation^10^ and likely associates with an autonomic induced response. Using short separation channels it was possible to extract the underlying brain response that is otherwise highly modulated by the sympathetic skin response.

We further observed that the response to noxious and innocuous electrical stimulation can be localized and distinguished over somatosensory cortex.

### Specificity of Response to Noxious Stimuli

The specificity of the response is supported by the following.

*First*, there is a temporal difference in the response to painful vs. non-painful stimuli over the S1 cortex. Such differences have been reported for both fMRI^14^,^17^,^18^ and NIRS^19^,^20^. In these studies, the response to such stimuli was mainly observed on the somatosensory cortex. The role of S1 is well defined in nociceptive processing although other areas including the anterior insular cortex, prefrontal cortices and thalamus are also involved^9^.

*Second*, there is a habituation of the brain response to noxious stimuli but not to innocuous stimuli. Habituation is the progressive decrease in response to a repetitive stimulus^21^. Studies of habituation during thermal stimuli^22^ have demonstrated decreased activity over time in anterior cingulate (ACC), insula (Ins), and primary (S1) and secondary (S2) somatosensory cortices. Recently, it has been demonstrated with electrical stimulation^23^ and similarly for thermal noxious habituation that ACC, Ins, S1, S2, as well as prefrontal cortex and cerebellum demonstrated habituation. Our results show no significant decrease over time in the hemodynamic response to innocuous stimuli, however some individual subjects did exhibit this in accordance with previous literature, which have reported habituation resulting from various non-painful stimuli. Examples include the visual system^24^, the frontal cortex during attention tasks^25^, and repetitive motor tasks^26^.

The underlying mechanism of habituation to repetitive painful stimuli is still unclear. Nociceptors, the sensory receptors at the primary afferent fiber terminals, transform noxious stimuli (either mechanical, chemical or thermal) into electrical signals and these are then transferred to the central nervous system. The input from ascending primary afferent neurons, interneurons and descending modulatory pathways interact at the dorsal horn to determine whether the signal will be transmitted to the brain through secondary afferent neurons. The pain transmission to higher cortical regions is inhibited via descending inhibition from higher brain regions that contain opioid receptors and endogenous opioids. If the painful stimuli cannot be avoided due to physical reasons or in favor of a superior goal, these endogenous antinociception mechanisms can be activated via cognitive centers in the brain^27^ and play an important role in habituation to painful stimuli. Supporting this, an increase in activity has been found in rostral anterior cingulate cortex (rACC), an endogenous pain control center, while other regions of the brain showed habituation^22^,^27^.

While habituation in the healthy condition is a normal physiological response, in certain pain conditions habituation is not present. For example, there is lack of habituation to painful stimuli in people with migraine attacks^28^,^29^ and chronic back pain^30^, and fibromyalgia^31^ patients have shown reduced habituation in response to electrical stimuli. It has been suggested that the lack of habituation may “contribute to the persistence of chronic pain”^29^. Drugs can alter the habituation response^32^. Evidence for morphine ‘dishabituation’ in response to pain has also been observed^33^. Taken together the use of habituation to stimuli can be evaluated in the context of disease state and processes that include central sensitization^34^ or centralization of pain^35^.

*Third*, noxious stimuli produced a bilateral response (the contralateral side showed a stronger brain activity as compared to ipsilateral side) while innocuous produced only a contralateral response. Such responses with noxious stimuli were also observed in S1 by previous studies^18^,^19^,^20^,^36^ while only contralateral responses were observed in other studies^37^,^38^. The differences could be arising from differences in experimental methodologies. In this study, under similar experimental and analytical conditions, noxious stimuli induced responses that were temporally and spatially different from innocuous ones.

### Pain response in the frontal region

Both innocuous and noxious stimuli resulted in a significant deactivation, that is a decrease in HbO concentration, in the superior and middle frontal gyrus, which was also previously shown with fNIRS by different groups^39^,^40^. Our results also show that the deactivation is stronger in response to noxious stimuli, though not statistically significant. Interestingly, the difference between the two stimuli becomes statistically significant between the [10–14] sec time range (paired t-test, p = 0.05 for HbO). Similarly the HbR change in response to noxious stimuli is larger in the second three minute period as compared to the first three minute period in a late time range [6–12] sec compared to stimulus presentation ([0–5] sec) (paired t-test, p = 0.003). This implies that pain also results in a late response which has to be further investigated with a larger group size, and a larger frontal probe.

### Caveats

Recent work by Holper et al. showed that painful stimulus results in a decrease in end tidal CO_2_ levels due to pain related hyperventilation^39^. This is a potential confounding factor since CO_2_ is a known vasodilator, and a decrease in end tidal CO_2_ should result in vasoconstriction. As we did not measure end-tidal CO_2_ in our experiments, we do not know if the stimulus we used caused a decrease in end-tidal CO_2_. However, if there is such an effect, any global change in the scalp is dealt with the short separation regression. Further, any CO_2_ induced change in the brain would produce a non-localized response, while instead we observed a localized response.

The conclusions from this study should further be tested on women subjects as there can be a gender related difference in brain response to pain^41^,^42^. Another future study is to test whether similar results would be observed with measurements separated by a time period allowing for reversal of the habituation. This would assure that repeated measurements during surgery would still give reliable results.

## Conclusion

In this work, we have shown that the painful and non-painful stimuli could be segregated and that repetitive noxious stimuli resulted in a measurable signal in the S1 region that showed specificity of location and function (habituation, altered level of response to noxious vs. non-noxious stimuli; and a signal that was distinguishable from a skin sympathetic response to pain). Our results support the notion that fNIRS has a strong potential to be used as an objective measure of pain in a fast and reliable fashion. In this study, we used fNIRS to investigate if repeated electrical innocuous and noxious electrical stimulation resulted in habituation of the response. Given that repeated electrical stimulation does not induce skin sensitization as thermal stimulation could, these results will help delineate the potential utility of noxious electrical stimulation to assess response modulation under variable analgesic levels.

## Methods

The study was approved by the Institutional Review Board of the Massachusetts General Hospital and met the scientific and ethical guidelines for human pain research of the Helsinki Accord and the International Association for the Study of Pain (http://www.wma.net/en/30publications/10policies/b3/). The methods used in this study were carried out in accordance with the guidelines approved by Institutional Review Board of the Massachusetts General Hospital.

### Probe and System

**Figure 7:**
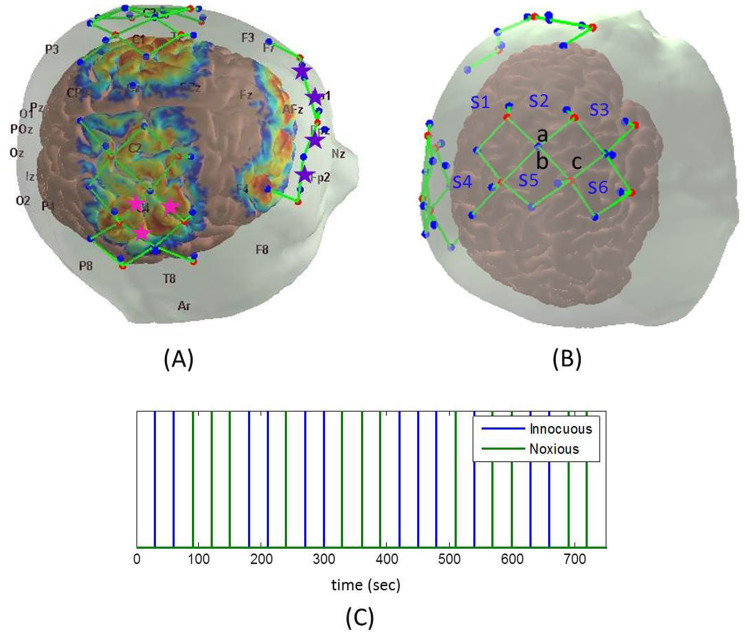
Probe placement and stimulus paradigm. The locations of the detectors (blue dots), the sources (red dots), the channels (green lines) and the sensitivity profile are shown for one subject (panel A). The optodes and the channels are displayed on the scalp and the sensitivity profile is displayed on the cortex. The sensitivity profile is displayed on a logarithmic scale and spans two orders of magnitude (arbitrary units). The channels used for frontal (purple) and motor-sensory (pink) regions are labelled with stars. The letter-number combinations on panel B (long separation channels: a, b, c and short separation channels: s1 to s6) correspond to the same letter-number combinations on Figure 1, Figure 6 and Table 1. Panel C: Stimulus paradigm: innocuous stimuli (blue lines), noxious stimuli (green lines).

Data were collected using a multichannel imager operating at 690 and 830 nm (TechEn Inc. MA, USA, CW6 System). The probe contained 11 sources and 16 detectors (~3 cm distance from the source “long separation detectors”) and 11 short separation detectors (~8 mm distance from each source) (Figure 7, panel A and B). The long separation channels are sensitive to the brain and superficial layers (the scalp and the skull) while the short separation channels are sensitive to superficial layers only. The probe covers the somatosensory and motor areas as well as the frontal cortex.

### Subjects and Experimental Design

Eleven healthy subjects were included in the study (right handed, male, 28 ± 5 (mean ± std) years old). Each subject gave informed written consent prior to the experiments. Subjects with a history of neurological trauma or psychiatric disorders, or who were unable to keep their head still, were excluded.

Prior to the actual experiment, electrical stimulation was applied to each subject's left thumb through electrodes with a 5 Hz electrical stimulator (Neurometer CPT, Neurotron, Baltimore, MD) to determine current levels that elicited subjective ratings of 3/10 (innocuous) and 7/10 (noxious) from each subject. The electrical stimulus was increased from baseline (0.7 mA) by 0.05 mA increments, while the subjects were expected to specify when the electrical stimulus reaches to a pain level of 3 (innocuous) and 7 (noxious) on a 0 to 10 scale. These current values were used in the actual experiment.

During the actual experiment, randomized innocuous and noxious electrical stimuli at 5 Hz were applied by a neurometer (Neurotron Inc. Baltimore, MD). Each stimulus lasted 5 seconds, followed by a 25 second rest. Each run lasted 12.5 minutes and consisted of 12 innocuous and 12 noxious stimuli which were randomly ordered (Figure 7, panel C).

Following the NIRS acquisition, the 3D positions of the sources and detectors were obtained using a 3D digitizer (Polhemus Inc., VT).

### Data Analysis and Statistics

The raw NIRS signal was first converted into changes in optical density by taking the logarithm of the signal. The channels lower than 80 dB and higher than 140 dB were excluded from the analysis. The changes in oxygenated-hemoglobin (HbO) and deoxygenated-hemoglobin (HbR) concentrations were then obtained using the modified Beer-Lambert law with a partial pathlength factor of 6^4^,^5^,^6^. Motion artifacts were detected using hmrMotionArtifact function under HOMER2^43^ and the trials that have motion artifacts within −2 to 15 second window were excluded before the trial averaging analysis. A total of six trials were lost out of 66 trials across the 11 subject. The hemodynamic response function (HRF) was then estimated by a general linear model approach that uses ordinary least squares. The response was modeled using consecutive Gaussian temporal basis functions with a standard deviation of 0.5 second and their means separated by 0.5 second over the regression time range of −2 to 20 seconds as we have used previously in Ref. 44. The short separation channel with the highest correlation with a given long separation channel was used as a static estimator and regressed out from the long distance channel while simultaneously estimating the HRF as in Gagnon et al., 2011^44^. The method makes the assumption that the signal measured at the short separation channel represents the superficial layers and the signal measured at the long separation channel represents both brain tissue and superficial layers. Thus using short separation channels, the effect of systemic physiology can be captured from superficial layers and then can be used as regressors to filter systemic interference from the long separation channels to provide a more robust estimation of the underlying hemodynamic response to brain activation. Analysis was carried out using the open source software HOMER2, which is implemented in Matlab (Mathworks, Natick, MA).

T-tests were used to determine statistically significant differences in hemodynamic responses to innocuous and noxious stimuli in the first three minutes of acquisition as well as significant differences in the hemodynamic response in the first three minutes as compared to the second three minutes. The channels used and the time range averaged for these t-tests are shown in Figures 1 to 5. The channels used for the motor-sensory and the frontal region analysis are labelled with pink and purple stars respectively in Figure 7, panel A.

## Author Contributions

M.A.Y., C.A., D.B., D.A.B. and L.B. designed the research, and M.A.Y., C.A. and M.P. performed the experiments. M.A.Y., C.A., D.B., D.A.B. and L.B. analyzed the data. M.A.Y. prepared the figures, and M.A.Y., D.B., D.A.B. and L.B. wrote the manuscript. M.A.Y., C.A., M.P., D.B., D.A.B. and L.B. reviewed the manuscript.

## Figures and Tables

**Table 1:** The MNI coordinates of the midpoint of the source detector (SD) pairs of interest highlighted in Figure 1 and displayed on Figure 7, Panel B

channel	MNI coordinates	Location
a	50 -5 54	Right pre-central gyrus
b	48 -18 50	Right post-central gyrus
c	41 -19 43	Right post-central gyrus

## References

[b1] Jöbsis F. F. Noninvasive, infrared monitoring of cerebral and myocardial oxygen sufficiency and circulatory parameters. Science 198, 1264–1267 (1977). 929199 10.1126/science.929199

[b2] Boas D. A., Elwell C. E., Ferrari M. & Taga G. Twenty years of functional near-infrared spectroscopy: introduction for the special issue. Neuroimage 85 Pt 1, 1–5 (2014). 24321364 10.1016/j.neuroimage.2013.11.033

[b3] Scholkmann F. *et al.* A review on continuous wave functional near-infrared spectroscopy and imaging instrumentation and methodology. Neuroimage 85 Pt 1, 6–27 (2014). 23684868 10.1016/j.neuroimage.2013.05.004

[b4] Cope M. & Delpy D. T. System for long-term measurement of cerebral blood and tissue oxygenation on newborn infants by near infra-red transillumination. Med Biol Eng Comput 26, 289–294 (1988). 2855531 10.1007/BF02447083

[b5] Delpy D. T. *et al.* Estimation of optical pathlength through tissue from direct time of flight measurement. Phys Med Biol 33, 1433–1442 (1988). 3237772 10.1088/0031-9155/33/12/008

[b6] Boas D. A., Dale A. M. & Franceschini M. A. Diffuse optical imaging of brain activation: approaches to optimizing image sensitivity, resolution, and accuracy. Neuroimage 23 Suppl 1, S275–288 (2004). 15501097 10.1016/j.neuroimage.2004.07.011

[b7] Villringer A., Planck J., Hock C., Schleinkofer L. & Dirnagl U. Near infrared spectroscopy (NIRS): a new tool to study hemodynamic changes during activation of brain function in human adults. Neurosci. Lett. 154, 101–104 (1993). 8361619 10.1016/0304-3940(93)90181-j

[b8] Peyron R., Laurent B. & García-Larrea L. Functional imaging of brain responses to pain. A review and meta-analysis (2000). Neurophysiol Clin 30, 263–288 (2000). 11126640 10.1016/s0987-7053(00)00227-6

[b9] Apkarian A. V., Bushnell M. C., Treede R.-D. & Zubieta J.-K. Human brain mechanisms of pain perception and regulation in health and disease. Eur J Pain 9, 463–484 (2005). 15979027 10.1016/j.ejpain.2004.11.001

[b10] Nishimura T., Nakae A., Shibata M., Mashimo T. & Fujino Y. Age-related and sex-related changes in perfusion index in response to noxious electrical stimulation in healthy subjects. J Pain Res 7, 91–97 (2014). 24550680 10.2147/JPR.S57140 PMC3926458

[b11] Alkire M. T., White N. S., Hsieh R. & Haier R. J. Dissociable brain activation responses to 5-Hz electrical pain stimulation: a high-field functional magnetic resonance imaging study. Anesthesiology 100, 939–946 (2004). 15087631 10.1097/00000542-200404000-00026

[b12] Becerra L. *et al.* Trigeminal neuropathic pain alters responses in CNS circuits to mechanical (brush) and thermal (cold and heat) stimuli. J. Neurosci. 26, 10646–10657 (2006). 17050704 10.1523/JNEUROSCI.2305-06.2006 PMC6674763

[b13] Maihöfner C., Handwerker H. O. & Birklein F. Functional imaging of allodynia in complex regional pain syndrome. Neurology 66, 711–717 (2006). 16534108 10.1212/01.wnl.0000200961.49114.39

[b14] Becerra L. R. *et al.* Human brain activation under controlled thermal stimulation and habituation to noxious heat: an fMRI study. Magn Reson Med 41, 1044–1057 (1999). 10332889 10.1002/(sici)1522-2594(199905)41:5<1044::aid-mrm25>3.0.co;2-m

[b15] Rainville P., Bao Q. V. H. & Chrétien P. Pain-related emotions modulate experimental pain perception and autonomic responses. Pain 118, 306–318 (2005). 16289802 10.1016/j.pain.2005.08.022

[b16] Tsuzuki D. *et al.* Virtual spatial registration of stand-alone fNIRS data to MNI space. Neuroimage 34, 1506–1518 (2007). 17207638 10.1016/j.neuroimage.2006.10.043

[b17] Chang C. & Shyu B. C. A fMRI study of brain activations during non-noxious and noxious electrical stimulation of the sciatic nerve of rats. Brain Res. 897, 71–81 (2001). 11282360 10.1016/s0006-8993(01)02094-7

[b18] Chen J.-I., Ha B., Bushnell M. C., Pike B. & Duncan G. H. Differentiating noxious- and innocuous-related activation of human somatosensory cortices using temporal analysis of fMRI. J. Neurophysiol. 88, 464–474 (2002). 12091568 10.1152/jn.2002.88.1.464

[b19] Becerra L. *et al.* Diffuse optical tomography of pain and tactile stimulation: activation in cortical sensory and emotional systems. Neuroimage 41, 252–259 (2008). 18394924 10.1016/j.neuroimage.2008.01.047 PMC2728450

[b20] Becerra L. *et al.* Diffuse optical tomography activation in the somatosensory cortex: specific activation by painful vs. non-painful thermal stimuli. PLoS ONE 4, e8016 (2009). 19956637 10.1371/journal.pone.0008016 PMC2778627

[b21] Glaser E. M. & Whittow G. C. Evidence for a non-specific mechanism of habituation. J. Physiol. (Lond.) 122, 43–44P (1953). 13109800

[b22] Mobascher A. *et al.* Brain activation patterns underlying fast habituation to painful laser stimuli. Int J Psychophysiol 75, 16–24 (2010). 19833154 10.1016/j.ijpsycho.2009.10.008

[b23] Nickel F. T. *et al.* Brain correlates of short-term habituation to repetitive electrical noxious stimulation. Eur J Pain 18, 56–66 (2014). 23720364 10.1002/j.1532-2149.2013.00339.x

[b24] Obrig H. *et al.* Habituation of the visually evoked potential and its vascular response: implications for neurovascular coupling in the healthy adult. Neuroimage 17, 1–18 (2002). 12482064 10.1006/nimg.2002.1177

[b25] Butti M. *et al.* Effect of prolonged stimulation on cerebral hemodynamic: a time-resolved fNIRS study. Med Phys 36, 4103–4114 (2009). 19810483 10.1118/1.3190557

[b26] Shibuya K. The activity of the primary motor cortex ipsilateral to the exercising hand decreases during repetitive handgrip exercise. Physiol Meas 32, 1929–1939 (2011). 22048722 10.1088/0967-3334/32/12/004

[b27] Bingel U., Schoell E., Herken W., Büchel C. & May A. Habituation to painful stimulation involves the antinociceptive system. Pain 131, 21–30 (2007). 17258858 10.1016/j.pain.2006.12.005

[b28] De Tommaso M. *et al.* Lack of habituation of nociceptive evoked responses and pain sensitivity during migraine attack. Clin Neurophysiol 116, 1254–1264 (2005). 15978487 10.1016/j.clinph.2005.02.018

[b29] Valeriani M. *et al.* Reduced habituation to experimental pain in migraine patients: a CO(2) laser evoked potential study. Pain 105, 57–64 (2003). 14499420 10.1016/s0304-3959(03)00137-4

[b30] Flor H., Diers M. & Birbaumer N. Peripheral and electrocortical responses to painful and non-painful stimulation in chronic pain patients, tension headache patients and healthy controls. Neurosci. Lett. 361, 147–150 (2004). 15135915 10.1016/j.neulet.2003.12.064

[b31] De Tommaso M. *et al.* Laser-evoked potentials habituation in fibromyalgia. J Pain 12, 116–124 (2011). 20685171 10.1016/j.jpain.2010.06.004

[b32] Di Clemente L. *et al.* Topiramate modulates habituation in migraine: evidences from nociceptive responses elicited by laser evoked potentials. J Headache Pain 14, 25 (2013). 23566208 10.1186/1129-2377-14-25 PMC3620432

[b33] Cade L. & Ashley J. Towards optimal analgesia after caesarean section: comparison of epidural and intravenous patient-controlled opioid analgesia. Anaesth Intensive Care 21, 696–699 (1993). 8273900 10.1177/0310057X9302100537

[b34] Woolf C. J. Central sensitization: implications for the diagnosis and treatment of pain. Pain 152, S2–15 (2011). 20961685 10.1016/j.pain.2010.09.030 PMC3268359

[b35] Borsook D., Kussman B. D., George E., Becerra L. R. & Burke D. W. Surgically induced neuropathic pain: understanding the perioperative process. Ann. Surg. 257, 403–412 (2013). 23059501 10.1097/SLA.0b013e3182701a7b PMC3546123

[b36] Bosshard S. C. *et al.* Assessment of brain responses to innocuous and noxious electrical forepaw stimulation in mice using BOLD fMRI. Pain 151, 655–663 (2010). 20851520 10.1016/j.pain.2010.08.025

[b37] Shih Y.-Y. I., Wey H.-Y., De La Garza B. H. & Duong T. Q. Striatal and cortical BOLD, blood flow, blood volume, oxygen consumption, and glucose consumption changes in noxious forepaw electrical stimulation. J. Cereb. Blood Flow Metab. 31, 832–841 (2011). 20940730 10.1038/jcbfm.2010.173 PMC3063626

[b38] Disbrow E., Buonocore M., Antognini J., Carstens E. & Rowley H. A. Somatosensory cortex: a comparison of the response to noxious thermal, mechanical, and electrical stimuli using functional magnetic resonance imaging. Hum Brain Mapp 6, 150–159 (1998). 9673670 10.1002/(SICI)1097-0193(1998)6:3<150::AID-HBM4>3.0.CO;2-2 PMC6873382

[b39] Sakuma *et al.* Experimental pain in the gingiva and its impact on prefrontal corticalhemodynamics: A functional near-infrared spectroscopy study. Neuroscience Letters 575, 74–79 (2014). 24878385 10.1016/j.neulet.2014.05.040

[b40] Holper L. *et al.* Physiological effects of mechanical pain stimulation at the lower back measured by functional near-infrared spectroscopy and capnography. Journal of Integrative Neuroscience 13, 121–142 (2014). 24738542 10.1142/S0219635214500071

[b41] Ge H.-Y., Madeleine P. & Arendt-Nielsen L. Gender differences in pain modulation evoked by repeated injections of glutamate into the human trapezius muscle. Pain 113, 134–140 (2005). 15621373 10.1016/j.pain.2004.09.041

[b42] Wang G., Erpelding N. & Davis K. D. Sex differences in connectivity of the subgenual anterior cingulate cortex. Pain 155, 755–763 (2014). 24434729 10.1016/j.pain.2014.01.005

[b43] Huppert T. J., Diamond S. G., Franceschini M. A. & Boas D. A. HomER: a review of time-series analysis methods for near-infrared spectroscopy of the brain. Appl Opt 48, D280–298 (2009). 19340120 10.1364/ao.48.00d280 PMC2761652

[b44] Gagnon L. *et al.* Improved recovery of the hemodynamic response in diffuse optical imaging using short optode separations and state-space modeling. Neuroimage 56, 1362–1371 (2011). 21385616 10.1016/j.neuroimage.2011.03.001 PMC3085546

